# Supplementary material for: Novel Self-Forming Nanosized DDS Particles for BNCT: Utilizing A Hydrophobic Boron Cluster and Its Molecular Glue Effect
Source: Cells. 2022 Oct 21;11(20):3307. doi: 10.3390/cells11203307 (PMC9600043; doi:10.3390/cells11203307)
Supplement: Supplementary file 1 [file cells-11-03307-s001.zip › cells-1970127-supplementary.pdf]

Supporting information

Novel Self-forming Nanosized DDS Particles for BNCT: Utilizing A Hydrophobic Boron Cluster and its Molecular Glue Effect

Abdul Basith Fithroni[1, 2], Kazuko Kobayashi[3], Hirotaka Uji[4], Manabu Ishimoto[5], Masaru Akehi[3], Takashi Ohtsuki[1], and Eiji Matsuura[2, 3, 6, *]

[1] Department of Interdisciplinary Science and Engineering in Health Systems, Okayama University, 700-8530, Okayama, Japan
[2] Department of Cell Chemistry, Graduate School of Medicine, Dentistry, and Pharmaceutical Sciences, Okayama University, 700-8558, Okayama, Japan
[3] Collaborative Research Center for OMIC, Graduate School of Medicine, Dentistry, and Pharmaceutical Sciences, Okayama University, 700-8558, Okayama, Japan
[4] Department of Material Chemistry, Graduate School of Engineering, Kyoto University, 615-8510, Kyoto, Japan
[5] Fukushima SiC Applied Engineering Inc. 979-0513, Naraha-machi, Japan
[6] Neutron Therapy Research Center (NTRC), Okayama University, 2-5-1 Shikata-cho, Kita-ku, 700-8558, Okayama, Japan

* Correspondence: eijimatu@md.okayama-u.ac.jp (E.M.); Tel.: +81-86-235-7402 (E.M.)

List of contents

Figure S1. Features of the particles in each preparatory process for the AB-Lac particles loaded with diC12-Carb.

Figure S2. Transmission electron microscopy (TEM) images of the B compound-loaded AB-Lac particles.

Figure S3. *In vitro* 4T1 cell uptake of B amount (number) by incubating BPA or the AB-Lac particles loaded with *o*-carborane.

Table S1. Cytotoxicity of the AB-Lac particles loaded with carborane isomer or *o*-carborane alkylated derivative.

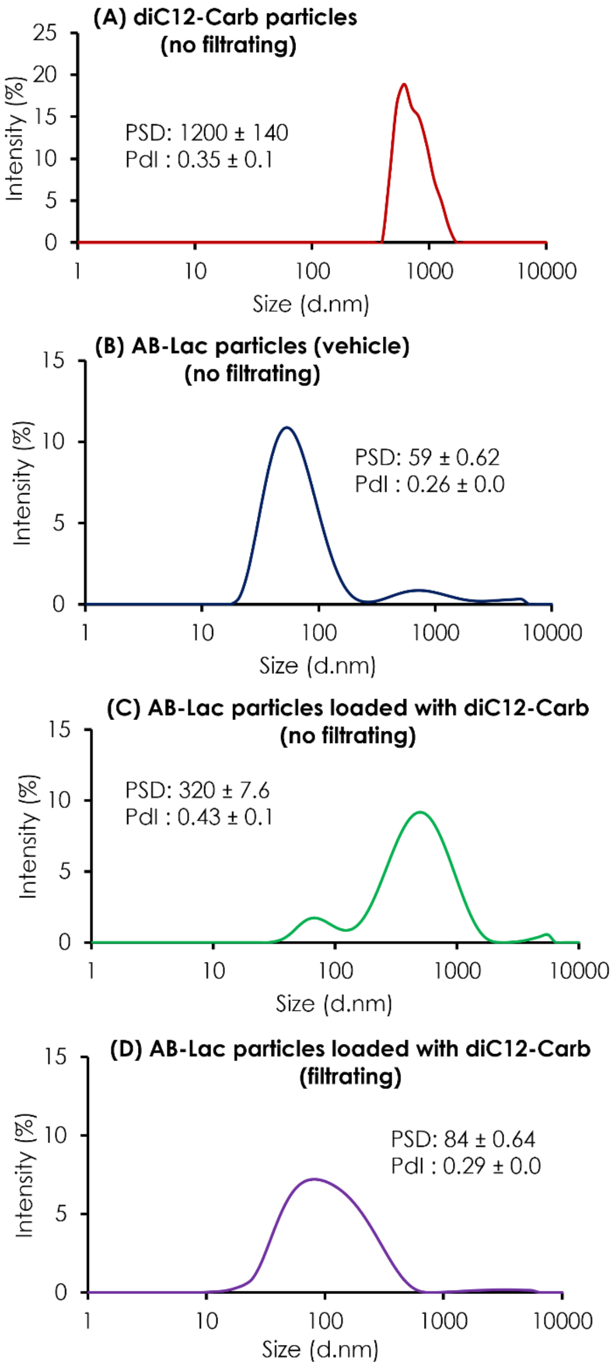

Figure S1. Features of the particles in each preparatory process for the AB-Lac particles loaded with diC12-Carb. Self-formed particles of diC12-Carb (without the AB-Lac polymers), after the passing through PD-10 but no filtrating (A), the AB-Lac particles (vehicle), after the passing through PD-10 but no filtrating (B), and the AB-Lac particles loaded with diC12-Carb, after passing through PD-10 but no filtrating (C), or the AB-Lac particles loaded with diC12-Carb, after the passing through PD-10 and filtrating (D). The filtration was performed after passing through the PD-10 column using 0.2 µm and 0.1 µm filters. The PSD and PdI are represented as mean ± S.D. (n=3). The **panels A-C** revealed a polydisperse pattern after the passing through the PD-10 column but no filtrating, large sized particles (aggregates) remained. Moreover, the data (**panel A**) also revealed that diC12-carb may form larger particles (aggregates) by themselves so that only a small amount of B was incorporated in the AB-Lac particles.

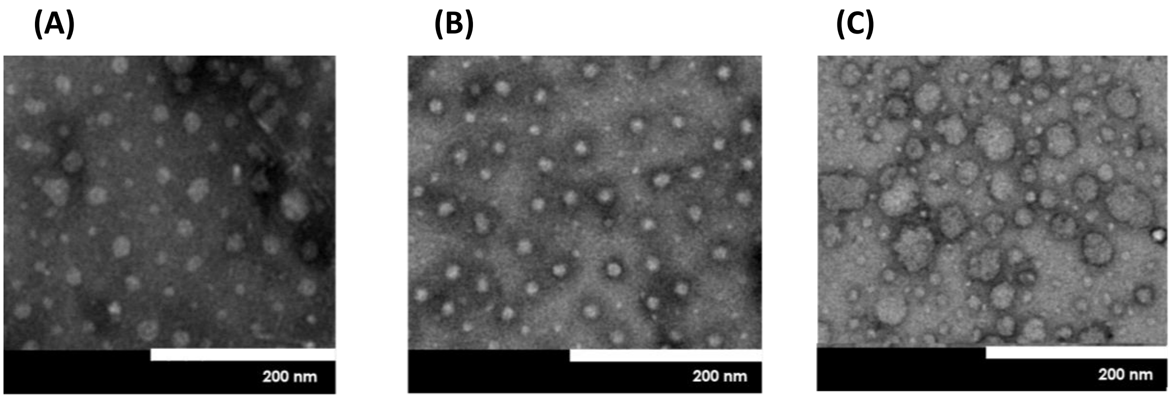

Figure S2. Transmission electron microscopy (TEM) images of the B compound-loaded AB-Lac particles. The AB-Lac particles (vehicle) (A), those loaded with *o*-carborane (B), or with diC6-Carb (C). The TEM images were obtained at an accelerated voltage, 80 kV, and magnification was 10,000x. These images confirmed that the PSD and PdI of the AB-Lac particles (vehicle) were almost like that of the particles loaded with *o*-carborane. The image also revealed that the particles loaded with diC6-Carb (C) were lager than others (A and B).

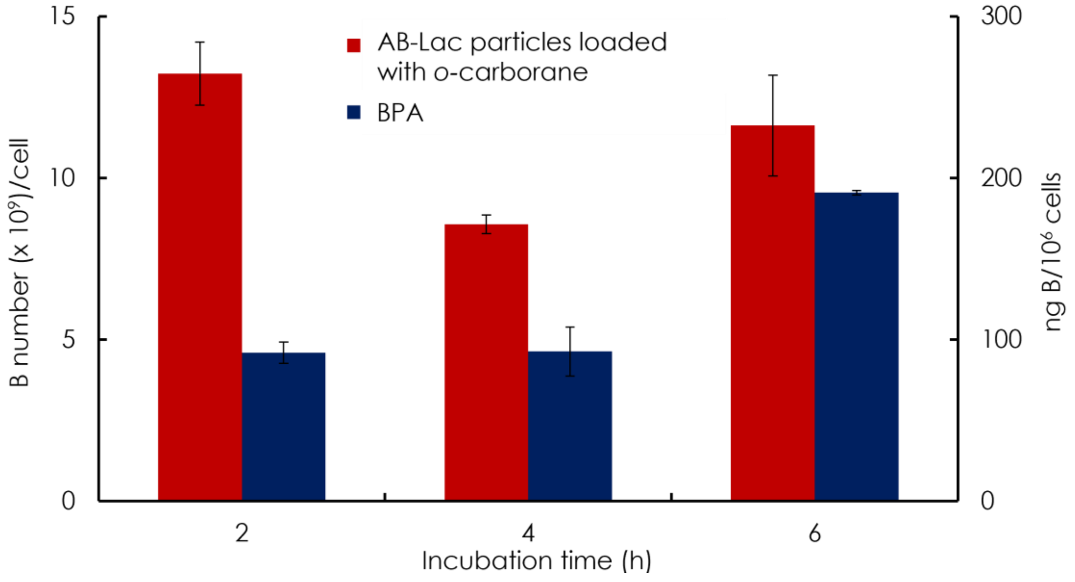

Figure S3. *In vitro* 4T1 cell uptake of B number (amount) by incubating BPA or the AB-Lac particles loaded with *o*-carborane. Time-dependent *in vitro* 4T1 cell uptake of B number was detected at 2, 4, and 6 hr, by incubating with BPA or the AB-Lac particles loaded with *o*-carborane (at 2 mM B equivalent). Data are expressed as mean ± SEM (n=3). Overall, the results showed that cell uptake of B from the AB-Lac particles loaded with *o*-carborane was higher than that of BPA. The AB-Lac particles loaded with *o*-carborane are taken up by any cancer cell lines probably by endocytosis. Conversely, the BPA is actively taken up by their L-amino acid transporter-1 (LAT-1) which is overexpressed in cancer cells.

Table S1. Cytotoxicity of the AB-Lac particles loaded with carborane isomer or *o*-carborane alkylated derivative to the 4T1 cells.

Carborane isomer/ *o*-carborane derivative	Cell viability (%) at			
	25 ppm	50 ppm	100 ppm	250 ppm
AB-Lac particles loaded with				
o-Carborane	130 ± 17	100 ± 4.6	99 ± 11	99 ± 8.0
m-Carborane	110 ± 14	97 ± 7.3	100 ± 5.1	110 ± 6.9
diC1-Carb	120 ± 7.3	110 ± 11	100 ± 8.9	93 ± 5.7
diC6-Carb	100 ± 3.2	97 ± 3.5	98± 2.7	95 ± 15

The cell viability was assayed by the CCK-8 kit, according to the kit's instructions. Data are expressed as mean ± SEM (n=4). The results showed no cytotoxicity at lower than 250 ppm.
